# Foams with 3D Spatially Programmed Mechanics Enabled by Autonomous Active Learning on Viscous Thread Printing

**DOI:** 10.1002/advs.202408062

**Published:** 2024-09-27

**Authors:** Brett Emery, Kelsey L. Snapp, Daniel Revier, Vivek Sarkar, Masa Nakura, Keith A. Brown, Jeffrey Ian Lipton

**Affiliations:** ^1^ Department of Mechanical and Industrial Engineering Northeastern University 815 Columbus Ave Boston MA 02120 USA; ^2^ Department of Mechanical Engineering Boston University 110 Cummington Mall College of Engineering Boston MA 02215 USA; ^3^ Department of Computer Science and Engineering University of Washington 185 E Stevens Way NE Seattle WA 98195 USA

**Keywords:** 3d printing, cellular structures, foams, self‐driving labs, viscous thread instability

## Abstract

Foams are versatile by nature and ubiquitous in a wide range of applications, including padding, insulation, and acoustic dampening. Previous work established that foams 3D printed via Viscous Thread Printing (VTP) can in principle combine the flexibility of 3D printing with the mechanical properties of conventional foams. However, the generality of prior work is limited due to the lack of predictable process‐property relationships. In this work, a self‐driving lab is utilized that combines automated experimentation with machine learning to identify a processing subspace in which dimensionally consistent materials are produced using VTP with spatially programmable mechanical properties. In carrying out this process, an underlying self‐stabilizing characteristic of VTP layer thickness is discovered as an important feature for its extension to new materials and systems. Several complex exemplars are constructed to illustrate the newly enabled capabilities of foams produced via VTP, including 1D gradient rectangular slabs, 2D localized stiffness zones on an insole orthotic and living hinges, and programmed 3D deformation via a cable‐driven humanoid hand. Predictive mapping models are developed and validated for both thermoplastic polyurethane (TPU) and polylactic acid (PLA) filaments, suggesting the ability to train a model for any material suitable for material extrusion (ME) 3D printing.

## Graphical abstract

This study introduces a method for 3D printing foams with predictable, spatially programmed mechanical properties using Viscous Thread Printing (VTP). The approach enables the creation of complex gradients and local property control. Predictive models are developed using a self‐driving lab for thermoplastic polyurethane and polylactic acid showcasing the mechanical and material versatility of VTP in expanding foam capabilities.

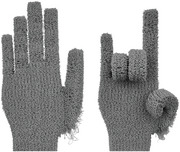

## 1 Introduction

Foams are ubiquitous for a wide range of common applications ranging from upholstery, packaging, and personal protective equipment, as well as more exotic functions such as medical implants, vibration attenuation, and structural lightweighting.^[^ ^1^, ^2^, ^3^ ^]^ Many of these applications primarily use homogeneous foams due to the prevalence of chemical manufacturing techniques used to produce large blocks of material.^[^ ^2^, ^3^, ^4^, ^5^, ^6^, ^7^, ^8^, ^9^, ^10^ ^]^ When a single uniform foam is unable to meet design requirements, the conventional approach is to rely on adhesive based lamination. This process is consequently restricted to creating discrete boundaries between property zones, resulting in composites with stress concentrations and discontinuities. In contrast, spatially resolved manufacturing processes such as 3D printing are not restricted by these limitations and can create more seamless transitions. In both natural systems and high‐performance engineering, continuous gradients are essential for establishing strong interfaces between hard and soft materials and for tailoring local mechanical responses.^[^ ^11^ ^]^ Despite their potential, existing methods for fabricating highly architected materials with complex continuous gradients have faced challenges related to performance and manufacturability, owing to the inherent complexity of these materials. This work introduces a method to 3D print complex gradients and localize property control in foam material space, characteristics traditionally considered incompatible with foam materials.

Material extrusion (ME) is a common form of 3D printing in which a material, such as a thermoplastic, is deposited layer by layer according to a prescribed geometry in order to form a volumetric part. A key feature of ME is the ability to spatially vary mechanical properties by patterning the underlying microstructure of an object. This is used for applications such as acoustic/elastic cloaking, patterning metamaterial mechanisms, personalizing biomechanical devices, etc.^[^ ^12^, ^13^, ^14^, ^15^, ^16^ ^]^ This ability to locally control material properties offers a unique approach to improving the functionality and manufacturability of foams. However, previous ME processes have struggled to manufacture foams due to various fundamental challenges.

ME‐produced foams have previously relied on three methods: 1) using secondary foaming materials, 2) directly injecting gas to produce voids, or 3) explicitly programming cellular geometries. Each method results in a limited range of pore sizes and stiffnesses, which then restricts the ability to spatially vary properties,^[^ ^7^, ^17^, ^18^, ^19^, ^20^ ^]^ as well as other process specific limitations. Foaming materials are constrained by the composition of chemical gassing/blowing agents or physical porogen inclusions (i.e., Syntactic Foaming^[^ ^20^ ^]^), limiting material compatibility and pore size control. This inflexibility in varying properties across foams makes it primarily suitable for producing large foam monoliths or hybridizing with other foam production processes.^[^ ^7^, ^18^, ^20^, ^21^ ^]^ Bubble‐based ME processes such as direct foam writing^[^ ^10^ ^]^ and direct bubble writing^[^ ^17^ ^]^ enable individual cell characteristics to be tailored locally while maximizing material usage efficiency. However, bubble‐based processes remain limited in material selection and lack fine detail resolution. Finally, for explicit cell geometry, the printer's resolution must be finer than the cellular structure's unit cell, typically by an order of magnitude or more,^[^ ^19^, ^22^, ^23^ ^]^ and requires architected void space which often results in isolated “islands” of structural material or internal sacrificial supports within each layer. These design constraints introduce the need for substantial travel and retraction moves or support material, reducing average structural mass flow rate, extending fabrication time, increasing manufacturing complexity, and escalating the probability of failures, particularly for sensitive materials such as soft or flexible feedstock; all of which limit applicability.

In comparison, viscous thread printing (VTP) leverages the physical phenomenon of viscous thread instability to produce open cellular materials without explicit cell design or complex pathing algorithms.^[^ ^24^ ^]^ VTP uses the dynamics of the printing process to control the microstructure of the object and consequently the underlying mechanical properties.^[^ ^24^, ^25^, ^26^ ^]^ As such, VTP is capable of exploiting the maximum volumetric flow rate of a particular material or printer to continuously produce voided cells at resolutions finer than the native resolution of the printer, without idle travel or retraction moves.^[^ ^27^, ^28^, ^29^, ^30^ ^]^ Existing work using similar and precursor methods to VTP has established its viability with various materials for soft robotic gripping,^[^ ^31^ ^]^ pressure sensing and proprioception,^[^ ^32^ ^]^ lightweight circuitry,^[^ ^33^ ^]^ food printing,^[^ ^34^ ^]^ architecture,^[^ ^35^, ^36^ ^]^ and artistic expression.^[^ ^37^, ^38^ ^]^ Our previous work with VTP created open‐cell foams with homogenous and limited gradient Young's moduli using TPU on standard ME printers and demonstrated a reduction in print artifacts and boundary defects, as well as enhanced failure performance over the previous state of the art.^[^ ^24^, ^25^ ^]^ However, establishing the correlation between VTP inputs and resultant material properties has required iterative manual testing of each parameter combination to determine their respective material properties and did not result in generalizable or transferrable models. By producing foams via VTP, we avoid the need for intricate design files or extended fabrication times, preserve the full selection of ME‐compatible materials, expand the ability to locally program mechanical properties, and offer an extensive cell size range.

Here, we develop a predictive model that links input VTP parameters to output foam material properties. Through this model, we discover that VTP is a self‐stabilizing process. That self‐stabilizing nature produces a parametric subspace that yields homogeneous foams by accurately predicting structural properties like effective layer height with R^2^ = 0.980. This subspace is demonstrated to be exclusive to the material specific dataset from which it was derived. However, this work characterizes the subspace for two distinct materials suggesting the existence of such a space for any material compatible with VTP. We realized a Gaussian process regression (GPR) model able to accurately map VTP coiling parameters to resulting material stiffness by exploring and characterizing the available property space through a data‐driven approach. To do this, we employed a self‐driving lab (SDL), which uses automation and active learning to perform experiments in high‐dimensional space without human intervention.^[^ ^39^, ^40^ ^]^ This model was then validated using leave‐one‐out cross validation (LOOCV) to predict the effective modulus with an R^2^ value of 0.946. An additional GPR model, based on input VTP parameters, was developed from principal component analysis (PCA) of the full stress–strain curves of foams to more broadly predict their mechanical properties. These models allow the rapid development of homogenous foams with targeted material properties. By applying these models continuously, they additionally facilitate gradient foams where the properties intentionally and predictably vary in space. Several complex exemplars were constructed to illustrate the newly enabled capabilities of foams produced via VTP, including 1D discrete and linear gradients on rectangular slabs, 2D localized stiffness zones on an insole orthotic and living hinges, and programmed 3D deformation via a cable driven anthropomorphic hand.

## 2 Results and Discussion

### 2.1 VTP Process Description

**Figure 1:**
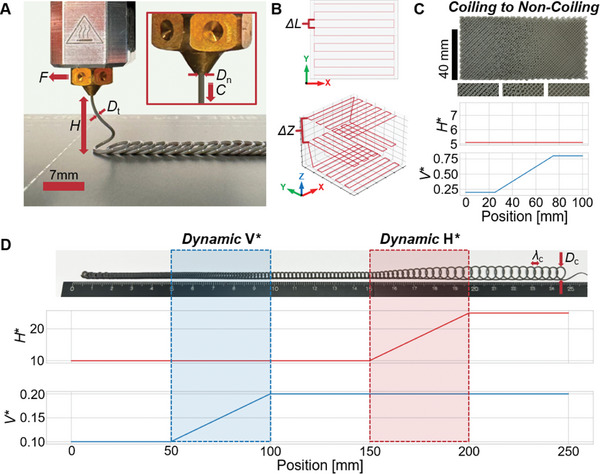
A) Typical material extrusion (ME) 3D printing setup during viscous thread printing (VTP), annotated with relevant parameters: nozzle translation speed (*F*), nozzle height (*Z*), thread diameter (*D*_t_), nozzle diameter (*D*_n_), and thread extrusion speed (*C*). B) 2D plot and 3D plot of a typical single‐layer and multi‐layer rectilinear toolpath, with annotated line spacing (*ΔL*) and layer spacing (*ΔZ*) respectively. C) Images of coiling behavior transitioning from coiling to non‐coiling ME, with close‐up images as seen on the left, middle, and right portions of the specimen, and corresponding plots of V^*^ and H^*^ as a function of position in x. D) 1D line of translating coiling with dynamic coiling behavior changes induced via V^*^ and H^*^ manipulation, annotated with coil wavelength (*λ*_c_) and coil diameter (*D*_c_), and corresponding H^*^ versus Position x and V^*^ versus x plots with respect to coiling behavior seen in C.

Fundamentally, VTP exploits the tendency of viscous liquids in the form of slender threads to coil upon deposition. This phenomenon, known as viscous thread instability (VTI), has been thoroughly characterized in 1D and 2D^[^ ^27^, ^28^, ^29^, ^30^, ^41^ ^]^ and is exemplified by the coiling pattern formed when honey is poured onto a surface. By applying this principle on conventional ME machines, a stationary nozzle produces consistent coiling behavior at scales smaller than the printer's native resolution. Furthermore, by following a simple linear toolpath, a continuous line of coils may be produced (**Figure** **1**A) which enables smooth and continuously varying material properties in space. This toolpath may be arranged in a simple rectilinear pattern in order to produce 2D meshes, which are then successively stacked to create 3D structures as seen in Figure 1B. The produced material is isotropic in the X‐Y plane while anisotropic in Z (see Figure S1, Supporting Information). The resulting open‐cell microstructure's geometry is stochastically distributed yet consistent with respect to its constituent coiling behavior, provided the printing conditions are maintained.

Two key parameters characterize VTP coiling behavior in 1D as described by:^[^ ^28^, ^30^, ^42^, ^43^ ^]^ the dimensionless velocity V^*^ and the dimensionless height H^*^, defined as:

(1) $$V^{*}=\frac{F}{C}$$

(2) $$H^{*}=\frac{H}{D_{t}}$$

where *F* is the translation speed of the printhead, *C* is the exit speed of the material from the nozzle, *H* is the height of the nozzle above the substrate, and *D*_t_ is thread diameter. The *D*_t_ is often larger than the nozzle diameter *D*_n_ due to die‐swell and can be calculated using the die‐swell parameter *α* using the equation *D*_t_ = *αD*_n_. Each of these variables is controlled either directly or indirectly through commands provided to the printer via G‐code, a numerical control programming language that provides precise instructions for the movements, speeds, and coordination of the print head and extruder during fabrication. Of the parameters which constitute V^*^ and H^*^, only *F* and *Z* are explicitly provided in G‐code. *C* may be calculated as a function of the linear filament feed rate due to the assumption that the filament volume flow into the nozzle is equal to the thread volume flow out of the nozzle,^[^ ^24^ ^]^ and *α* is a material‐ and process‐dependent constant that is determined empirically.

Maintaining control of coiling geometry is essential for realizing printable foams. Prior work has shown that *V* ^*^ is inversely proportional to linear coil density and therefore has a first order effect on coiling wavelength *λ*_c_, while *H* ^*^ is directly proportional to coil diameter *D*_c_ and has a second order effect on *λ*_c_.^[^ ^28^, ^30^, ^42^ ^]^ Coiling mode refers to the many types of distinct coiling patterns which may be produced by manipulating *V* ^*^ and *H* ^*^ such as translating coiling, alternating coiling, and equidimensional.^[^ ^28^, ^30^, ^42^ ^]^ Of these modes, translating coiling and alternating coiling represent the majority of useful outcomes, as they are the only two modes that produce consistent, self‐contained coils, which form the basis of the cellular structure characteristic of foams. However, the equidimensional mode also warrants consideration. Despite its lack of features resembling cellular structures, it serves as a bridge between VTP coiling and conventional ME non‐coiling processes as seen in Figure 1C. This is because both the equidimensional mode and conventional ME deposition occupy only the space directly beneath the nozzle along the prescribed toolpath. As such, the coiling mode parameters *V* ^*^ and *H* ^*^ have the most influence over microstructure which, beyond base material properties, is the primary driving factor behind the foams’ material properties. While *V* ^*^ and *H* ^*^ are the generally dominant variables for determining coiling behavior and resulting geometry, foundational studies concerning VTI have established the significant influence of material viscoelastic properties on coiling behavior.^[^ ^28^, ^37^, ^42^, ^43^ ^]^ As such, *V* ^*^ and *H* ^*^ should only be considered to have dominant influence over coiling behavior so long as base material and environmental factors are consistent.

By varying *V* ^*^ and *H* ^*^ as a function of space, we may continuously vary the coiling mode and therefore locally control microstructure (Figure 1C,D). In order to practically use the aforementioned coiling modes to produce a coherent volume, the extruding nozzle must follow a specified toolpath. For maximum generality, we have chosen to maintain a simple rectilinear toolpath with each consecutive layer being rotated 90° (Figure 1B). The shape of this rectilinear toolpath is determined by the variables *ΔL* and *ΔZ*, referring to the spacing of parallel lines and *Z*‐height increment of sequential layers, respectively. Both of these parameters have a significant influence on the overall properties of the resulting part as they dictate where the material is deposited. However, due to self‐interaction between deposited threads and preceding layers, predicting the exact geometry of the deposited thread and its aggregate structures is challenging. Consequently, determining the impact on the overall foam structure resulting from tuning any or all VTP parameters has traditionally necessitated explicit testing between iterations.

### 2.2 Modeling and Homogeneous Subspace

While the flexibility of VTP is a virtue from a materials design perspective, tuning the processing parameters to realize homogenous materials presents a challenge. Specifically, when the first layer of a sample is printed, the processing variables V^*^, H^*^, and *ΔL* collectively determine the layer height *H*_L_. For a VTP structure to be homogeneous, *ΔZ* must equal *H*_L_ so that the distance between the print nozzle and substrate does not change when printing the next layer, ensuring the same average microstructure from layer to layer. Therefore, for each V^*^, H^*^, and *ΔL* there is a unique *ΔZ* that will lead to a homogeneous foam. The subspace in which these conditions are met is referred to as the homogeneous subspace.

Previous works have explored methods such as computational simulation in an effort to characterize the coiling behavior observable within VTP. However, these works focused primarily within 0D or 1D, as such the 2D to 3D regimes remain largely computationally unexplored.^[^ ^28^, ^37^, ^42^, ^43^ ^]^ This is predominantly due to the computational intractability of systems capable of simulating the high number of degrees of freedom of interaction required to conduct finite element analysis on a stochastic microstructure over large deformations as is consistent with VTP, and are therefore susceptible to many sources of noise. This has led us to pursue Gaussian process regression (GPR) models based on compiled large datasets, as opposed to discrete finite element simulations from first principles. If a theoretical model to predict the coiling behavior of VTP in 3D is developed, incorporating it into the GPR model as an informed prior could improve both the accuracy of the model and the speed of learning, as shown in previous work on transfer learning.^[^ ^44^ ^]^

**Figure 2:**
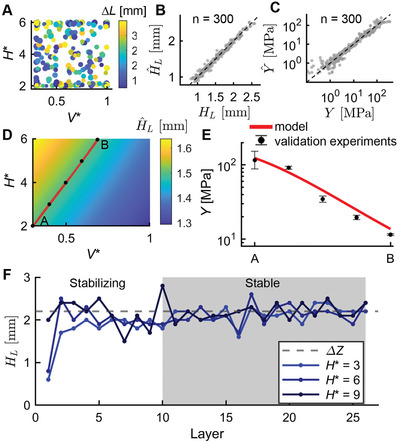
A) H^*^ versus V^*^ for all 300 experiments performed in polylactic acid (PLA) with color indicating *ΔL* and marker size indicating *ΔZ*. B) Parity plot of predicted layer height *Ĥ*_L_ versus layer height *H*_L_ for Gaussian process regression (GPR) model using leave‐oneout cross validation (LOOCV). C) Log‐log parity plot for predicted effective modulus *Ŷ* versus effective modulus *Y* for the GPR model using LOOCV. D) Slice in 2D of H^*^ and V^*^ where *ΔL* and *ΔZ* are equal to 1.5 mm. The color indicates *Ĥ*_L_ and the red line indicates the predicted homogenous subspace where *Ĥ*_L_ = *ΔZ*. Black dots indicate five equally spaced samples selected for validation testing. E) GPR model's *Ŷ* (red line) versus results of five validation experiments (black). Error bars represent one standard deviation in semi‐log space from each condition being tested in triplicate. F) *H*_L_ versus layer number for three PLA cubes with different H^*^. After several layers, *H*_L_ for each cube stabilizes to 2.2 mm, which is equal to *ΔZ*.

In order to predict the correct *ΔZ* for each V^*^, H^*^, and *ΔL* triplet, we used an SDL to run an experimental campaign that iteratively selected and performed 300 experiments using Bayesian optimization to minimize the uncertainty across the full 4D space on VTP foams formed out of polylactic acid (PLA) (**Figure** **2**A). After these experiments, the performance of the final GPR model to predict *H*_L_ was evaluated using leave‐one‐out cross validation (LOOCV) and found to have R^2^ = 0.980 (Figure 2B). In addition, we also conditioned a GPR to predict an effective modulus *Y* of each part, which was found to have a LOOCV R^2^ value of 0.946 (Figure 2C). Importantly, these two models can be combined to allow precise control of the properties of a homogenous cube by enabling inverse design to target a desired *Y*.

We hypothesized that the SDL‐derived models for *Ĥ*_L_ and *Ŷ* could allow predictive control over the VTP outcome and enable us to fully exploit the capabilities of VTP printing. In particular, a major goal is to spatially vary the mechanical properties of a material in a manner that is easy and reliable to fabricate. One approachable way to implement this would be to maintain a constant *ΔL* and *ΔZ* throughout the part, thus enabling a consistent rectilinear toolpath as shown in Figure 1B, while maintaining the utility of V^*^ and H^*^ to change the *Y* of different areas of the part. To explore this possibility, a 2D slice of the H^*^ and V^*^ parameter space at a constant *ΔL* and *ΔZ* can be visualized (Figure 2D). The *Ĥ*_L_ is shown, with the red line indicating the homogenous subspace for which *Ĥ*_L_ = *ΔZ*. Note that while this red line appears to be linear, the underlying GPR model does not assume linearity. Five points that are equally spaced from A to B are selected along this homogenous line. The *Y* of the physical experiments is then compared to the predicted effective modulus *Ŷ* from the GPR model, showing that it can be modulated between ≈10 and 100 MPa while remaining in the homogenous subspace with constant *ΔL* and *ΔZ* (Figure 2E).

While the presence of a smooth homogenous subspace that facilitates the manufacture of graded materials is a powerful outcome of this study, the shape of the inhomogeneous region also revealed a fortuitous relationship in the underlying VTP process. Specifically, Figure 2D shows that there is a positive correlation between H^*^ and *H*_L_. This positive correlation implies the existence of a stabilizing basin of attraction to correct errors in the selection of *ΔZ*. If *ΔZ* is too large, then *H*_L_ < *ΔZ*, and the effective H^*^ will increase each layer until *H*_L_ = *ΔZ*. In contrast, if *ΔZ* is too small, then *H*_L_ > *ΔZ*, and the effective H^*^ will decrease until *H*_L_ = *ΔZ*. Consequently, the component will homogenize at a new effective H^*^ if the selected parameters are not already in the homogenous subspace, although this process will change the properties of the foam and produce a non‐homogenous zone for several layers during stabilization. For this reason, it is important to determine the correct *ΔZ* before printing. Nevertheless, the basin of attraction means that small errors in the selection of *ΔZ* will not be catastrophic to the printing process and that any anomalies during printing, such as variations in *V* ^*^ caused by discrepancies in the filament diameter or variations in *H* ^*^ caused by the stochastic coiling of the VTP process at each layer, will self‐correct. To demonstrate this basin of attraction, we printed three samples with the same V^*^, *ΔL*, and *ΔZ* with variations in their *H* ^*^. After printing these samples, we measured the thickness of each layer. As expected, *H*_L_ for each of the experiments stabilized at the selected *ΔZ*, regardless of H^*^ (Figure 2F).

### 2.3 Dynamic Coiling and Gradient

**Figure 3:**
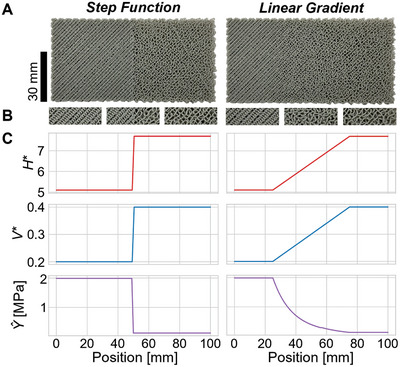
A) Rectangular specimens with constant layer heights, as modeled by the homogeneous subspace, demonstrating gradient coiling behavior. The specimens transition from high to low density from left to right, exhibiting both discrete and linear changes in coiling behavior respectively. B) Close‐up images of coiling behavior observed in the left, middle, and right portions of each specimen, respectively. C) Corresponding plots of H^*^, V^*^, and *Ŷ*, from top to bottom, as a function of position.

Having isolated the homogeneous subspace, we can query it to maximize the available property space of compatible homogeneous foams. While both *V* ^*^ and *H* ^*^ affect microstructure density, *V* ^*^ is the primary factor in determining linear coil density and, consequently, has the strongest impact on overall VTP foam density. As such, previous work was able to establish that continuous gradients could be achieved by singularly varying *V* ^*^.^[^ ^24^ ^]^ However, without also varying *H* ^*^ to maintain layer thickness homogeneity, the range of mechanical properties that can be achieved in these foams, without significant structural consequences, is severely limited. Only by applying the homogeneous subspace model pioneered by this work, is the full VTP mechanical property space able to be navigated without inducing unintentional layer thickness variations. Furthermore, the application of this model enables the ability to guarantee layer thickness compatibility between distinct microstructures. Thereby allowing for the programming of zones with specific desired microstructure dependent mechanical properties, and the creation of bespoke transition regions such as discrete boundaries or continuous gradients as seen in **Figure** **3**, without unintended structural consequences. These gradients are possible despite the self‐stabilizing nature of VTP because this characteristic of VTP behaves as a basin of attraction to a particular *ΔZ* for any given *V* ^*^, *H* ^*^, *ΔL*. As navigating the homogeneous subspace by definition maintains *ΔZ*, there is no significant resistance to a change in coiling behavior so long as *ΔZ* is maintained across transition regions regardless of orientation.

### 2.4 Exploration of Enabled Capabilities

**Figure 4:**
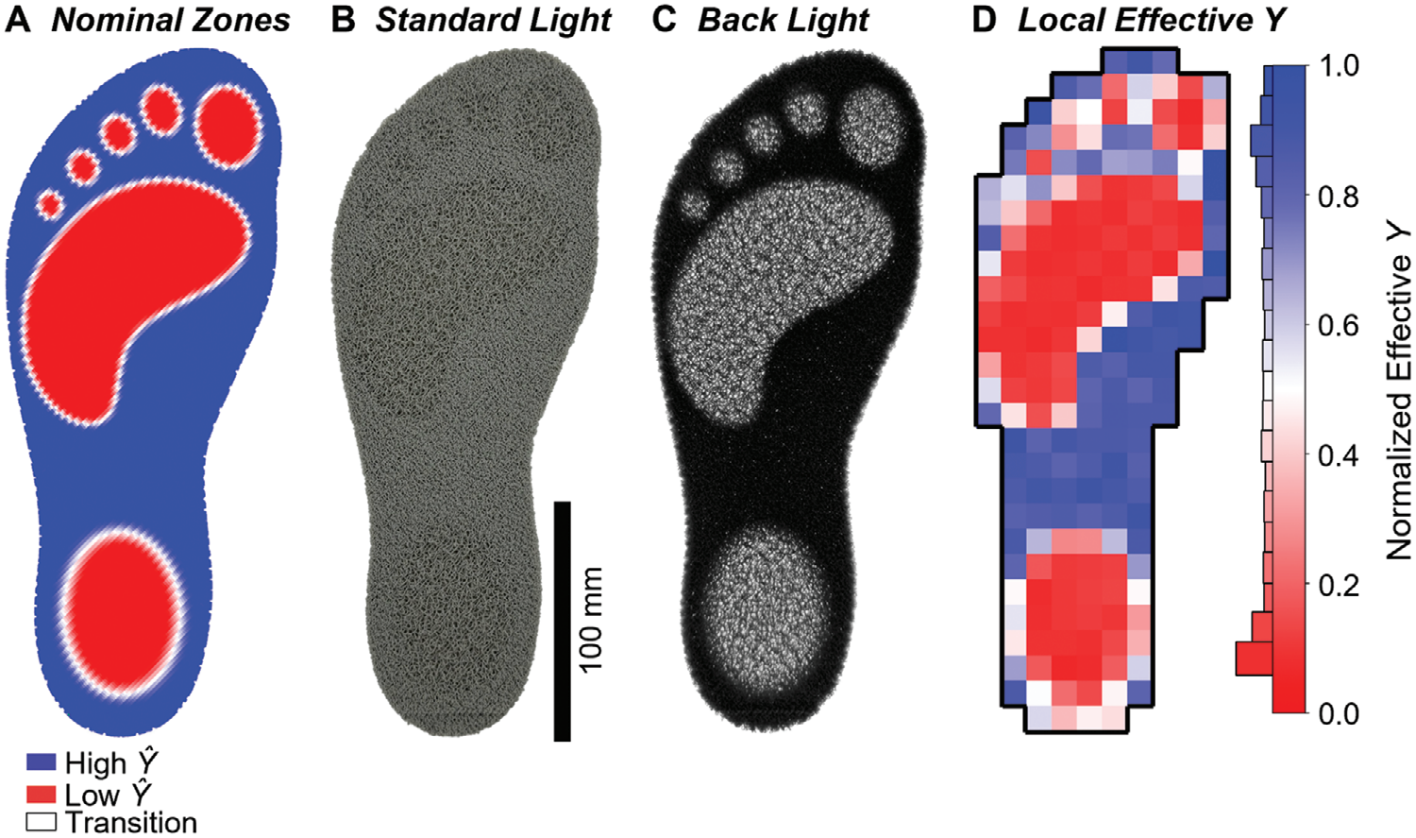
A) Density color coded toolpath of example multi‐stiffness VTP orthotic. B) Top view of printed orthotic under standard lighting conditions. C) Top view of printed orthotic when illuminated from beneath. D) Normalized local effective *Y* map of orthotic with respective color bar and occurrence histogram.

By applying gradients to induce specific output material properties, a wide variety of potential application spaces emerge. For example, fine control over compressive stiffness is vital to applications such as padding, cushioning, or pressure relieving apparatus. To explore whether VTP as a method to tailor stiffness could be used in these contexts, we designed and printed the custom orthotic seen in **Figure** **4**. We validated the stiffness of this orthotic by conducting localized compression tests. This generated the local effective *Y* map seen in Figure 4D. We normalized the modulus to highlight relative extremes in the modulus. Additionally, due to the relationship between microstructure, density, porosity, and stiffness in cellular materials^[^ ^2^ ^]^ for relatively thin objects, such as the orthotic in Figure 4, the effect of VTP programmed microstructure zones also manifests in the form of different surface textures as seen in Figure 4B, as well as transparency due to porosity in Figure 4C.

**Figure 5:**
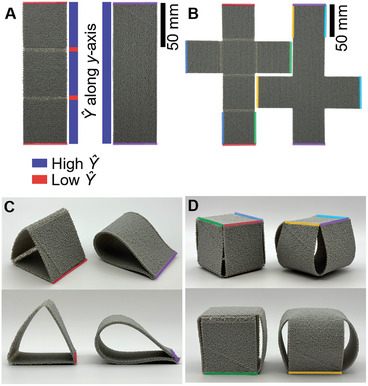
Examples of folding nets where the left column of each quadrant contains specimens with VTP compliant hinges, while the right is the same nets without hinges. Fixed edges are color coded respectively. A) Top view of unfolded triangular nets with annotated *Ŷ* zones along the *y*‐axis. B) Top view of unfolded box nets. C) Isometric and front views of folded triangular nets. D) Isometric and front views of folded box nets.

Programmed deformation is an additional capability enabled by programmed density and stiffness which we explore in both 2.5D and 3D objects. In existing literature programmed deformation in 2D, 2.5D, and 3D have been applied for use in examples such as metamaterial doorknob mechanisms and pliers,^[^ ^15^, ^45^ ^]^ as well as for use in soft robotics.^[^ ^32^ ^]^ Utilizing VTP's ability for programmed stiffness, we show that the local reduction of stiffness enables selective motion through elastic deformation in the form of compliant joints and hinges^[^ ^46^, ^47^ ^]^ as seen in **Figure** **5**. Due to the relative stiffness differences between adjacent zones, these examples illustrate how deformation may be proactively constrained to particular regions, thereby minimizing undesired deformation in higher stiffness areas. A geometric net refers to a 2D object that may be modified in order to form a 3D shape.^[^ ^48^ ^]^ Figure 5A shows a set of triangular geometric folding nets, while Figure 5B shows a set of folding box nets in both undeformed and deformed states. For the deformed states shown in Figure 5C,D states were achieved by affixing the appropriate free edges of the nets as seen in Figure 5A,B using fishing wire. No other external influences besides the edge‐to‐edge fixture and the stiffness zones were applied to alter the final state of these deformed nets. The curvature observable in both specimens with and without hinges is entirely due to boundary conditions applied over the flexible VTP material. The difference in stiffness within the included joints versus the adjacent material allows for a significant reduction in deformation of the higher stiffness faces, thus resulting in a greater agreement between the intended geometry and final geometry based on the undeformed net arrangement.

**Figure 6:**
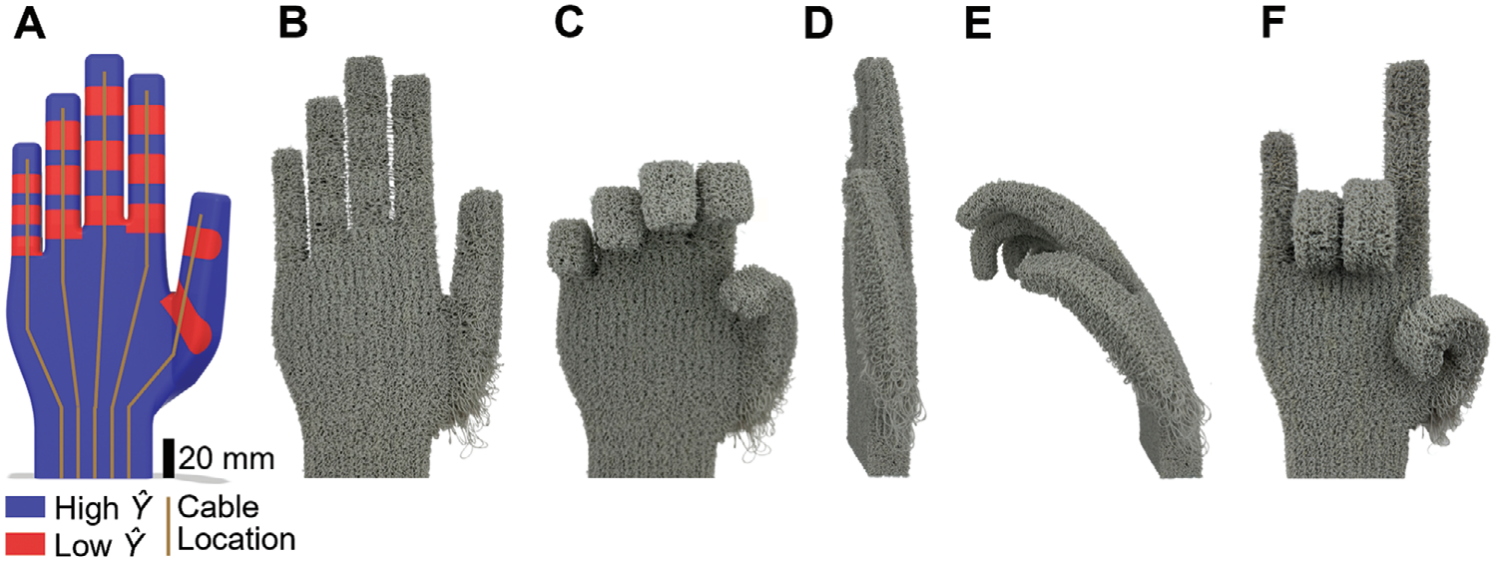
Example of VTP hand cable actuated with a fishing wire threaded along the surface of the palm and fingers utilizing *Ŷ* zones placed along typical joint locations for selective deformation. A) 3D model used to manufacture printed hand color coded according to assigned *Ŷ* zones and cable location on the surface of the palm. B) Front view of undeformed hand. C) Front view of partially deformed hand. D) Side view of undeformed hand. E) Side view of partially deformed hand, here the localized influence of lower stiffness to create joints may be most clearly observed via the non‐uniform bending of the index and middle fingers. F) Front view of selectively deformed hand in the form of a complex gesture.

Programmed VTP material properties can be applied volumetrically, enabling programmed 3D deformation, as seen in **Figure** **6**. Much like in thin 2.5D materials, volumetric regions of low stiffness preferentially deform and bend when adjacent to regions of higher stiffness material. By controlling the location and degree of this stiffness differential, VTP produces programmable deformation and bending within arbitrary geometry and conditions. As a demonstration, we created a foam approximation of a human hand as seen in Figure 6. The joints of the hand were achieved by lowering the stiffness of semi‐cylindrical zones at the location of each joint found in a typical hand (Figure 6A). A simple cable driven system capable of emulating a variety of gestures as seen in Figure 6B–F was then created by threading fishing wire up through the already porous surface of the palm and fingers. The approximate arrangement of these wires along the surface of the palm may be seen in Figure 6A. This system was able to individually contract each finger in a simple downward motion. Additional degrees of freedom may be enabled by adding additional cables along the external surface of the desired motion.

### 2.5 Common Foam Emulation

**Figure 7:**
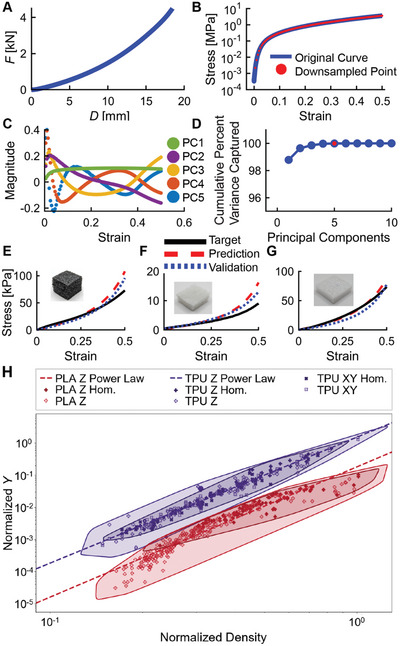
A) Force (*F*)–Displacement (*D*) of a TPU cube compressed to a 4.5 kN force limit. B) Log stress–strain curve (blue) converted from F–D curve is downsampled to 100 points (red) equally spaced to 50% strain. C) Principal component analysis breaks down sampled curves into 100 components (only the top five are shown for clarity). D) Cumulative percent variance captured by the number of components. The vast majority of variance is captured by the first five components. E–G) Stress–Strain curves for three foam samples (black curves), the sample predicted to most closely match their performance (red dashed), and the tested performance of that predicted sample (blue dotted). H) Ashby plot containing all relevant density and *Y* data collected on the SDL system for PLA and TPU normalized to the relevant properties of respective bulk material. Power‐laws of the homogeneous datasets yielded powers of 3.87 with an R^2^ of 0.75 for PLA when loaded in Z, 3.57 with an R^2^ of 0.97, and 3.62 with an R^2^ of 0.96 for TPU when loaded in *Z* and *X* respectively. This suggests a consistent trend of VTP foam behavior relative to bulk characteristics.

In addition to controlled deformations, we hypothesized that the processing freedom afforded by VTP would allow VTP foams to replicate the material performance of common foams. To explore this, we ran an additional campaign with the SDL to explore VTP print parameters for TPU filament. As before, the campaign used active learning to fully explore the parameter space with 155 experiments. Next, F‐D curves (**Figure** **7**A) were converted to stress–strain curves based on the sample geometry. The stress–strain curves were then down sampled to 100 stress points that were equally spaced in strain from ε = 0.005–0.5 (Figure 7B). We then took the logarithm of these stress values to preclude the possibility of predicting negative stress. Principal component analysis was then applied to all 155 TPU samples tested (Figure 7C,D). A separate GPR model was conditioned on the scores for each of the first five principal components as a function of their processing conditions. These GPR models could then be used to predict the performance of VTP foams that have not yet been tested.

In order to evaluate the VTP foam selection pipeline we selected three reference foam samples from everyday objects, consisting of a polystyrene (PS) foam used for packing/shipping (Figure 7E), a cellulose foam used for packing/shipping (Figure 7F), and a PS foam used as part of a children's car seat (Figure 7G). These foams were not selected due to any anticipated similarities with the characteristics of VTP foams, rather they were selected purely due to the ubiquitousness of their application. They were tested in quasi‐static compression using the same protocol as the VTP foams. The performance of each foam was then compared to the predictions of 125,000 potential VTP experiments selected using Latin hypercube sampling. An error function minimized the mean squared error of the difference between the log stress prediction and the foam stress. The VTP cube with the lowest error was selected and tested, showing the ability to match the performance of common foam samples. The ability of VTP foams to emulate conventional foams serves to emphasize that the VTP process may be employed to specifically target mechanical property spaces occupied by conventionally manufactured foams. Notably the stress–strain behavior being demonstrated closely aligns with elastomeric foam cellular structures within the elastic buckling plateau and densification as defined by Gibson, Lorna J. “Cellular Solids: Structure and Properties”.^[^ ^2^ ^]^

VTP manipulates microstructure and its dependent mechanical properties by introducing void space. Therefore, the bounds of the mechanical property space are directly related to the bounds of the porosity of the foam. As there is no theoretical maximum *H* ^*^, and consequently no theoretical maximum cell size, the only limit to how much the density of the material can be reduced from bulk is *D*_t_ as the minimum *H*_L_ and length scale separation of cells and the overall part. In order to fully visualize the expanded range of stiffnesses that VTP enables, as tested on the SDL system, a comprehensive Ashby plot was created for normalized modulus versus normalized density, relative to respective bulk material properties, as seen in Figure 7H. This plot reveals that, within the limited scope of the data collected, the range of moduli was extended downward from bulk by approximately three orders of magnitude for both PLA and TPU. Each homogeneous material dataset was fit to power laws of the form *y*  =  *x^B^*. When loaded along the Z and X axes TPU exhibited powers of 3.57 and 3.62 with corresponding R^2^ values of 0.97 and 0.96 respectively, while PLA in Z exhibited a power of 3.87 with an R^2^ of 0.75. The combined homogeneous datasets of both materials corresponded to a power of 3.70 with an R^2^ of 0.89. These ranges of power law orders differ from the expected powers of a purely bending or stretch‐dominated material.^[^ ^2^ ^]^ The results of this data suggest that VTP consistently alters the bulk behaviors of the printed material in a predictable manner. Furthermore, it is hypothesized that these regions do not represent the maximum achievable range of mechanical properties for these materials, as the data collected did not include all possible VTP parameter sets. Thus, further research may reveal a wider range of microstructures and their associated mechanical properties.

## 3 Conclusion

This work demonstrates a transformative way of spatially varying the material properties of foams using simple desktop printers. By harnessing viscous thread instability to enable VTP, we enable localized customization of mechanical properties by creating gradient microstructures. These gradients demonstrate spatially variable capabilities beyond what is currently achievable via conventional chemical foam manufacturing. Due to the correlation between microstructure and mechanical properties in cellular materials, the ability to spatially vary microstructure results in spatially variant mechanical properties, including localized stiffness, porosity, and deformation in 3D. This VTP process enabled a significantly expanded property space by deploying a predictive model that explores a newly discovered homogeneous subspace, linking input VTP parameters to output foam material properties without compromising structural intent. Additionally, the exploration of this subspace revealed an underlying self‐stabilizing characteristic of VTP layer thickness, allowing for an acceptable margin of error when selecting VTP characteristics that affect overall layer geometry. This model was produced using an SDL which combined automated experimentation with machine learning. The application of this model enabled us to replicate the stiffness of a selection of foams used in packaging, padding, and cushioning, suggesting that VTP may be used to emulate significant characteristics of commonly distributed conventional foams. The predictive mapping models were developed and validated for TPU and PLA, suggesting these models may be trained on any material compatible with ME 3D printing, while the results of the normalized mechanical property space relative to bulk suggest the effects of VTP on mechanical properties are consistent and predictable across materials. This body of work primarily focused on the characterization of the stiffness of materials manufactured via VTP. Therefore, in order to more comprehensively characterize the inherent properties of these materials, future areas of work include quantitative analysis of pore size distribution, cell geometry, environmental resilience, and cyclic mechanical testing. Overall, these findings indicate that VTP offers a versatile and precise method for manufacturing foams with tailored properties beyond the current state of the art, potentially paving the way for a wide variety of new application spaces for foams and 3D printed materials.

## 4 Experimental Section

### G‐Code Generation

G‐code files for 3D printing of test specimens and demonstration samples were generated using two different methods. For the test specimens, a Python program generated G‐code for a cube with a side length *L* and hardcoded a rectilinear infill. Parameters *V* ^*^, *H* ^*^, *ΔL*, *ΔZ*, and *α* were input and applied homogeneously, resulting in a uniform, cubic test specimen. The second method involved a custom slicer capable of processing an arbitrary number of non‐overlapping STL mesh files with varying geometries. This was used for all samples with inhomogeneous densities. Each imported mesh was assigned a local *V* ^*^/*H* ^*^ pair, while a global *ΔL*, *ΔZ*, and *α* were prescribed for the entire print. The slicer generated a G‐Code with a rectilinear infill for the union of all meshes, refined the toolpath, and assigned relevant G‐code values (*Z*, *E*, and *F*) based on the local *V* ^*^/*H* ^*^. This resulted in a single toolpath for arbitrary geometry with G‐Code parameters specified by the local *V* ^*^/*H* ^*^ values. In an effort to allow coiling behaviors to stabilize before entering the work volume, VTP deposition commenced as soon as the nozzle departs the printer's home coordinates. If the resulting line of coils connecting printer home coordinates with initial print coordinates overlaps with print volume, this will result in excess material buildup along this line. If the printed part is sufficiently thin, such as those seen in Figure 5, a distinctive ridging artifact will be visible on the top surface of the part.

### Layer Height Measurements

Layer height measurements (Figure 2F) were made through a manual process. First, a single rectangular prism 60 mm in height and with *L* = 30 mm was printed from PLA for *H* ^*^ = 3, *V* ^*^ = 0.3, *ΔL* = 1.5, and *ΔZ* = 2.2. The height of the cube was measured using a universal testing machine (UTM) (Instron 5965) by lowering the platen manually until the force increased to 1 N, ensuring that stray strands of filament did not artificially increase the height measurement. The height of the cube was taken as the platen separation of the UTM. Then, the bottom layer of the cube was removed with wire cutters. Because the filament was more tightly bonded within the layer than between layers, it was possible to cut away a single layer without disturbing the remaining layers. However, because of the thin nature of the removed layer, it was destroyed in the process. The height of the remaining cube was then measured in the UTM with the 1 N threshold. By computing the difference in height between the two measurements, the height of the removed layer was inferred. This process was repeated until only two layers remained. Unfortunately, because the final layers were so thin, measurement of the last two layers was not possible. This process was repeated for the other two cubes with *H* ^*^ = 6 and 9.

### Automated Testing

Automated testing leveraged the Bayesian experimental autonomous researcher (BEAR) previously developed.^[^ ^39^, ^40^ ^]^ The system consisted of a UTM (Instron 5965), a scale (Sartorius CP225D), a material extrusion (ME) printer (Prusa Mk3S+), and a six‐axis robot arm (Universal Robotics UR5e). The BEAR was a self‐driving lab (SDL), which leveraged the combination of active learning for experiment selection and automation for higher experimental throughput. This combination has been shown to improve not only grid‐based search methods but also subject matter experts.^[^ ^40^, ^49^, ^50^, ^51^ ^]^ The learning loop consisted of several steps performed in sequence. First, an experiment was selected using active learning. GPR models were conditioned in MATLAB using the built‐in function fitrgp using a squared exponential kernel with automatic relevance detection. Potential experiments were calculated using Latin hypercube sampling and an experiment was selected using a maximum variance decision‐making policy. Initially, *Y* was used as the target metric for the GPR, but it was later changed to *H*_L_ to ensure that both metrics were fully mapped.

G‐code for the selected experiment was then generated using a custom Python script. The G‐code was sent to the printer using OctoPrint through the Python package OctoRest. For PLA (eSun PLA+ gray), the printing temperature was set to 215 °C and the bed temperature was set to 60 °C. For TPU (Ninjatek Ninjaflex blue), the printing temperature was set to 230 °C and the bed temperature was set to 50 °C. For both materials, the part was removed by the robot after the bed had cooled below 32 °C.

The part was then transferred to the scale where it was weighed. If the measured mass was more than 5% of the expected value, the part was discarded due to the likelihood of a print error. After recording the mass, the part was transferred to the UTM for testing. During testing, the UTM lowered its platens at 2 mm min^−1^ until the force reached 4.5 kN or the platens were separated by less than 0.4 mm.

The modulus was calculated by converting the force–displacement curve into a stress–strain curve. The height for this conversion was measured by the UTM when the force exceeded 0.3 N. The cross‐sectional area was assumed to match the designed dimensions for the cube of 30 × 30 mm^2^. The modulus was then calculated using a linear fit of the stress between 5% and 15% strain to avoid any toe regions. Modulus normalization was performed by dividing the measured modulus of each specimen by the modulus of the corresponding spool of filament. Density normalization was calculated by first determining the measured density of each specimen by dividing its measured mass by volume and then dividing this value by the material density provided in the associated material technical data sheet. Specimen volume was determined using the measured height of the specimen, identified by the modulus force threshold, and multiplying it by the square of the nominal specimen width plus *ΔL*. This adjustment accounts for variations in side length due to differences in coil diameter from the specified toolpath.

The *H*_L_ of the part was calculated by taking the height of the final part and dividing it by the number of layers. For a part to be considered homogenous, *H*_L_ must be within 5% of *ΔZ*.

### Principal Component Analysis

The stress–strain curve was converted to log10 and truncated at 50% strain. It was then down sampled to 100 equally spaced points. PCA was performed using MATLAB's built in pca function. Because 99.99% of the cumulative variance was captured by the first five components, only the first five components were used for stress–strain curve prediction. For each of these components, a GPR model was conditioned to predict the score of the component based on the 4D input space (V^*^, H^*^, ΔL, and *ΔZ*). Sampling points (125,000) were then selected using Latin hypercube sampling for V^*^, H^*^, and *ΔL. ΔZ* was selected using the *H*_L_ GPR model to set *ΔZ* = *H*_L_, therefore ensuring that the resulting sample was homogenous. The predicted score components were then transformed back into the log10 stress/strain space. The predicted curves were compared to the foam samples using the error function shown in Equation 3. The closest curve was selected for each foam type and tested.

(3) $$Error=\frac{\sum {\left(\log\left({\hat{\sigma }}_{n}\right)-\log\left(\sigma_{n}\right)\right)}^{2}}{n}$$

Foam samples were cut into 30 × 30 mm^2^ rectangular prisms to match the cross‐section of the sample VTP foams, but the heights of the foam samples were not modified. The foams were weighed by hand and manually transferred to the UTM. The UTM tested them using the same 2 mm min^−1^ speed and the same 4.5 kN stop threshold protocol.

### Local Effective Stiffness Testing

For the orthotic stiffness heatmap as seen in Figure 4D, a and local effective Young's Modulus method was employed over 199 data points, evenly distributed 20 mm apart in both *X* and *Y* directions across the surface of the orthotic. This data was collected utilizing an Instron 68SC‐2 Universal Testing System equipped with a 2 kN load cell affixed with a flat circular 10 mm diameter probe printed on a Carbon M1 out of UMA‐90,^[^ ^23^ ^]^ mounted axially above a 10 kN testing anvil. The orthotic was printed on a standard Prusa Mk4 using NinjaTek NinjaFlex TPU. The resulting orthotic was ≈6 mm in thickness.

The procedure for testing included an initial 1 mm^−1^s^−1^ ramp until a force threshold of 1 N was met to establish the height of initial contact. The probe then returned 1 mm to pause for one‐half second before starting data collection at 6 mm min^−1^ for a total of 2 mm deformation.

The local effective modulus was calculated by converting the force–displacement curve into a stress–strain curve. The term “local effective modulus” refers to the fact that this testing method used a probe that did not fully envelope the cross‐section of the orthotic to sample the average mechanical stiffness of a small localized region of the overall part. The cross‐sectional area was assumed to match the designed dimensions of the probe of π x 5^2^ mm^2^. Young's modulus was calculated using a linear fit of the stress–strain data between 10 and 15% strain to avoid any toe regions. Since the material being tested in this method was not isolated from the surrounding material, it was expected that the resulting data would be influenced by boundary effects.

## Conflict of Interest

The authors have a patent application for path planning methods used (Patent Application 63/527,296 filed 7/17/2023).

## Supporting information



Supporting Information

## Data Availability

The data that support the findings of this study are openly available in VTP‐BEAR at https://bit.ly/VTP‐Data, reference number 0.
