# Supplementary material for: Foams with 3D Spatially Programmed Mechanics Enabled by Autonomous Active Learning on Viscous Thread Printing
Source: Adv Sci (Weinh). 2024 Sep 27;11(44):2408062. doi: 10.1002/advs.202408062 (PMC11600193; doi:10.1002/advs.202408062)
Supplement: Supplementary file 1 — Supporting Information [file ADVS-11-2408062-s001.docx]

Supporting Information

**Foams with 3D Spatially Programmed Mechanics Enabled by Autonomous Active Learning on Viscous Thread Printing**

*Brett Emery†^^[[1]](#footnote-1)^^ Orcid: 0000-0002-3163-225X*

*Kelsey L. Snapp† Orcid: 0000-0001-5984-0723*

*Daniel Revier Orcid: 0000-0001-6246-7819*

*Vivek Sarkar Orcid: 0009-0000-4120-8973*

*Masa Nakura Orcid: 0009-0000-6210-1702*

*Keith A. Brown Orcid: 0000-0002-2379-2018*

*Jeffrey Ian Lipton* Orcid:0000-0003-0843-0999*

*Affiliations*

B. A. Emery, J. I. Lipton*

Department of Mechanical and Industrial Engineering, Northeastern University, 815 Columbus Ave, Boston, MA 02120, United States

E-mail: [emery.b@northeastern.edu](mailto:emery.b@northeastern.edu), [j.lipton@northeastern.edu](mailto:j.lipton@northeastern.edu)

K. L. Snapp, K. A. Brown

Department of Mechanical Engineering, Boston University, 110 Cummington Mall College of Engineering, Boston, MA 02215, United States

E-mail: [ksnapp@bu.edu](mailto:ksnapp@bu.edu), [brownka@bu.edu](mailto:brownka@bu.edu)

D. Revier, V. Sarkar, M. Nakura

Department of Computer Science and Engineering, University of Washington, 185 E Stevens Way NE, Seattle, WA 98195, United States

E-mail: [drevier@cs.washington.edu](mailto:drevier@cs.washington.edu), [viveksar@uw.edu](mailto:viveksar@uw.edu), [mnakura@cs.washington.edu](mailto:mnakura@cs.washington.edu)


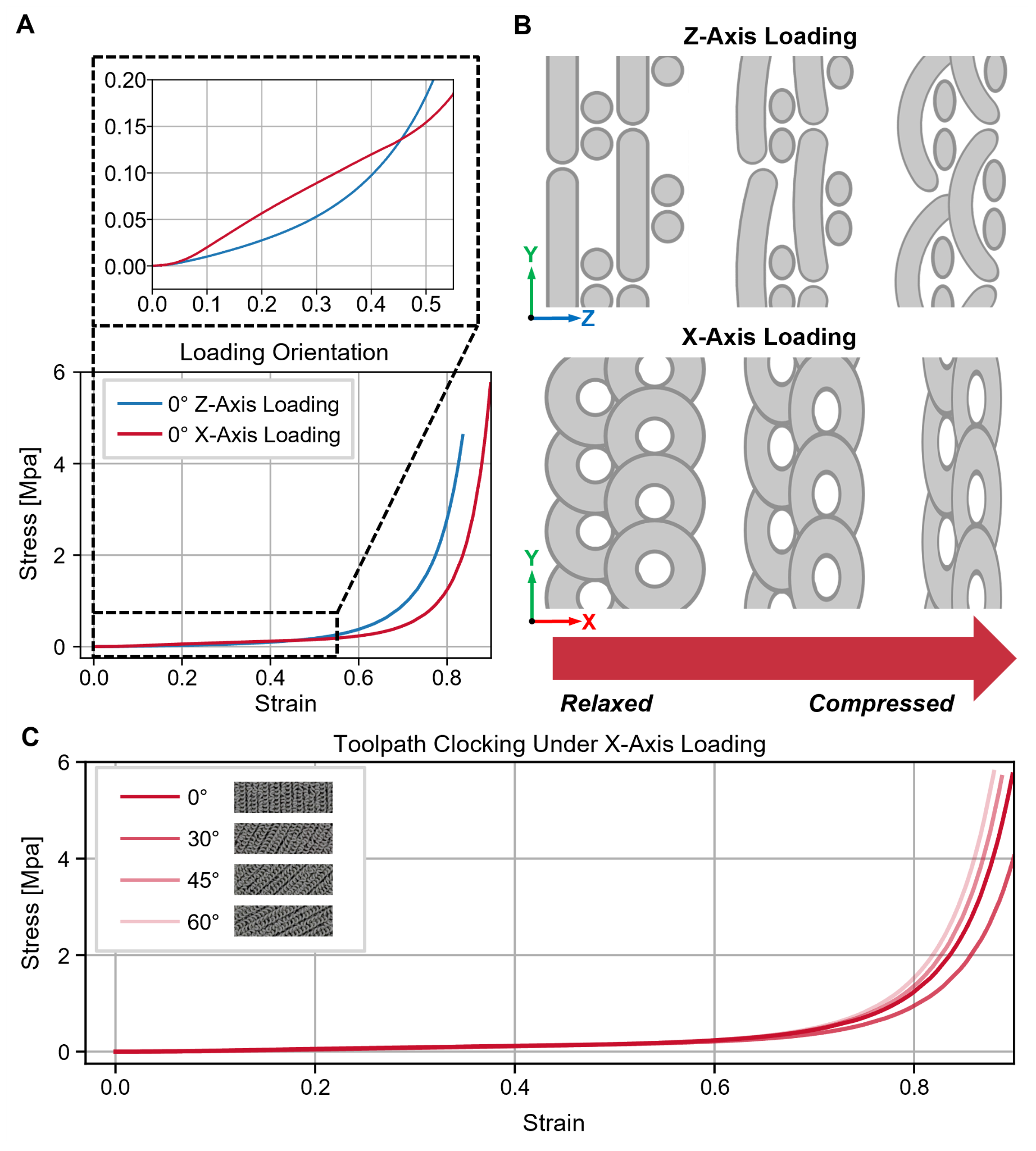


**Figure S1.** A) Plots depicting stress-strain curves of representative average density TPU specimens tested in compression along the X and Z axes to examine the response of in-plane and out-of-plane loading, respectively. Under X-axis loading a more distinct linear elastic region is visible relative to Z-axis loading resulting in Young’s moduli values of 0.33MPa and 0.17MPa respectively. This difference in behavior is likely the result of the difference in microstructure along the Z and X directions. B) Schematic illustration of deformation behavior under in-plane and out-of-plane loading. C) Plot depicting stress-strain curves of specimens fabricated with 0°, 30°, 45°, and 60° toolpath angle clocking offset under X-axis loading. These curves are very closely aligned under large deformations resulting in each specimen adhering to a Young’s moduli value of 0.33MPa, suggesting that VTP foams are highly isotropic within the XY plane.

In an effort to validate the mechanical property behavior of VTP foams, additional compression testing was conducted on specimens along both Z and X axes, and on specimens with various toolpath clocking angles. The results of supplemental Figure 1A show that VTP foams exhibit subtly different behaviors when tested in Z or XY, and therefore these foams should be considered anisotropic in Z. Supplemental Figure 1B, depicts a schematic representation of the differential deformation mechanics of a VTP structure under Z-axis loading compared to X-axis or Y-axis loading due to the unique cell geometry of VTP structures. However, supplemental Figure 1C demonstrates that regardless of the clocking of the toolpath, the resulting foam is isotropic in the XY plane. This isotropy is evident not only in the extracted Young's modulus but also in the shape of the stress-strain curve up to the point of densification.

Received: ((will be filled in by the editorial staff))
Revised: ((will be filled in by the editorial staff))
Published online: ((will be filled in by the editorial staff))

1. † Co-first authors [↑](#footnote-ref-1)
